# Nanomaterials for Removal of Phenolic Derivatives from Water Systems: Progress and Future Outlooks

**DOI:** 10.3390/molecules28186568

**Published:** 2023-09-11

**Authors:** Maricely Ramírez-Hernández, Jordan Cox, Belvin Thomas, Tewodros Asefa

**Affiliations:** 1 Department of Chemical and Biochemical Engineering, Rutgers, The State University of New Jersey, 98 Brett Road, Piscataway, NJ 08854, USA; 2 Department of Chemistry and Chemical Biology, Rutgers, New Brunswick, Rutgers, The State University of New Jersey, 98 Brett Road, Piscataway, NJ 08854, USA

**Keywords:** photocatalysis, mesoporous materials, triclosan, phenolic compounds, emerging pollutants, multifunctional nanomaterials

## Abstract

Environmental pollution remains one of the most challenging problems facing society worldwide. Much of the problem has been caused by human activities and increased usage of various useful chemical agents that inadvertently find their way into the environment. Triclosan (TCS) and related phenolic compounds and derivatives belong to one class of such chemical agents. In this work, we provide a mini review of these emerging pollutants and an outlook on the state-of-the-art in nanostructured adsorbents and photocatalysts, especially nanostructured materials, that are being developed to address the problems associated with these environmental pollutants worldwide. Of note, the unique properties, structures, and compositions of mesoporous nanomaterials for the removal and decontamination of phenolic compounds and derivatives are discussed. These materials have a great ability to scavenge, adsorb, and even photocatalyze the decomposition of these compounds to mitigate/prevent their possible harmful effects on the environment. By designing and synthesizing them using silica and titania, which are easier to produce, effective adsorbents and photocatalysts that can mitigate the problems caused by TCS and its related phenolic derivatives in the environment could be fabricated. These topics, along with the authors’ remarks, are also discussed in this review.

## 1. Introduction

**Figure 1:**
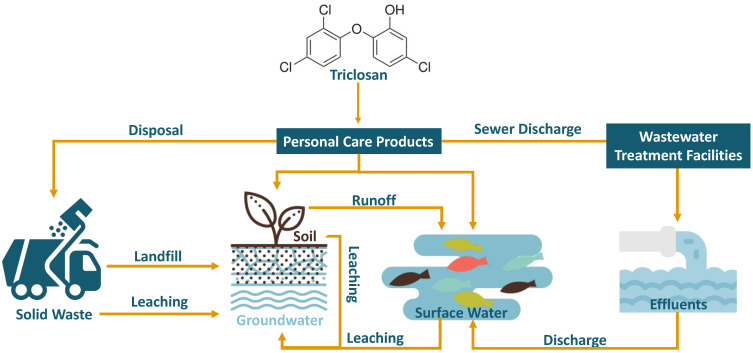
Some of the major sources of triclosan (TCS) and ways by which it can enter water systems and the environment.

In the next several decades, 68% of the world’s population is expected to live in cities that are highly dependent on water sources affected by agricultural runoffs and industrial and municipal wastewater discharges [1]. Triclosan (TCS or 2,4,4′-trichloro-2′-hydroxydiphenyl ether) is a widely used preservative and antimicrobial agent in personal care products such as soaps, skin creams, toothpaste, and deodorants as well as in household items such as plastic chopping boards, sports equipment, and shoes. Its mild nature without compromised efficacy has made it a widely accepted and commonly used active ingredient for these applications worldwide [2]. However, the prevalence of this chlorinated phenolic compound and its derivatives has started to present concerns to aquatic life as the majority of TCS and other phenolic derivatives enter the sewage systems and waste treatment plants (Figure 1). Because of TCS’s lipophilicity, with an octanol-water partition coefficient of 4.8 [3], and thus its propensity for organic particles [4], the discharge of effluents and sludge from wastewater treatment plants comprise the most prominent source of TCS in the aquatic environment [5]. As a result of pressure from the public, regulation, and policy, TCS has been banned from being used as an over-the-counter product by agencies such as the United States Federal Drug Administration (FDA) in 2016. Similarly, consumer product companies have also begun phasing out the ingredient from many of their products.

However, as of this year, the FDA is not proposing clinical outcome studies for active ingredients when used in healthcare antiseptics or consumer antiseptic rub products. The FDA requires clinical simulation studies for healthcare antiseptics because of ethical concerns with conducting studies in the healthcare setting. The FDA also requires clinical simulation studies for consumer antiseptic rubs because they are intended for use when soap and water are not available. Therefore, they need not demonstrate clinical effectiveness compared to soap and water. Meanwhile, in many other parts of the world, TCS is regulated to not exceed specific concentrations in consumer products such as cosmetic preservatives, textiles, plastics, and adhesives (see Table 1). This has particularly become important as recent reports have shown that TCS is a potential endocrine disruptor and has effects on the placental endocrine function in pregnant rats [6,7,8,9,10,11]. TCS does so mainly by inhibiting the circulating steroid hormone production and altering the expression of hormone metabolism enzyme genes in the placenta, thereby compromising fetal development and growth.

**Figure 2:**
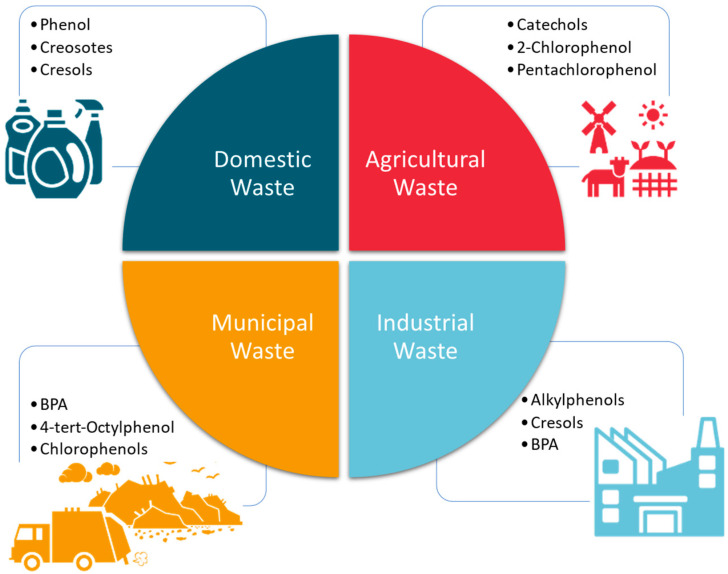
Some phenolic compounds that are related to TCS and that are used in various consumer products are shown. These are also increasingly becoming emerging environmental pollutants worldwide.

In addition to TCS, several other phenolic compounds are used as broad-spectrum antimicrobials, additives in surface cleaners, and precursors for many industrial processes (pharmaceuticals, surfactants, and pesticides). They include cresols, catechols, bisphenol A (BPA), and nonylphenol ethoxylates. Similar to TCS, these compounds are becoming emerging pollutants because of their widespread use as well as their endocrine-disrupting capabilities. They have thus been listed by various agencies, such as the United States Environmental Protection Agency (US EPA) and the European Union (EU), as pollutants of priority concern. The most notable of these groups of compounds is BPA. Figure 2 summarizes the types and sources of common phenolic compounds that are increasingly becoming emerging environmental pollutants.

Meanwhile, research on nanomaterials with potential applications for environmental remediation has been ongoing over the last several years because these materials have a great ability to adsorb and sometimes photocatalyze various chemical contaminants. For example, inorganic nanomaterials composed of silica and titania can be synthesized with large surface areas to serve as adsorbents of TCS and related phenolic compounds and to minimize or eliminate the negative impacts of these compounds and their derivatives in aquatic environments. Moreover, inorganic materials composed of semiconducting properties, such as titania, exhibit photocatalytic activities to degrade these compounds.

TCS is one of the seven most frequently detected phenolic compounds in the streams in the US, with varying concentrations ranging from ng/L to low µg/L [17,18]. TCS is also found in surface water and soils across the world. TCS with concentrations of 20–13,300 µg/kg in discharged biosolids [19] and 200–2700 ng/L in effluents leaving wastewater treatment plants in different places are documented as well [18]. In the environment, TCS with concentrations of 1.4–40,000 ng/L in lakes, rivers, and surface water [7,19]; 580–15,600 µg/kg in activated, digested sludge [7,19]; and up to 800 ng/kg in freshwater are recorded [18]. TCS has become ubiquitous in the environment in large part due to discharge from various sources associated with consumer products and its ability to infiltrate water systems as a result of its heavy use by consumers. This has been exacerbated by the recent COVID-19 pandemic, which has increased the demand for phenolic compounds to make various cleaning and other consumer products, with a compounded annual growth rate (CAGR) of approximately 4% [15,20].

The toxicity of TCS has been studied in numerous animal models, with interest dedicated to its effect on soil and aquatic ecosystem health. TCS has raised concern because high concentrations of this emerging pollutant can disrupt the balance of the ecosystem by killing microalgal species, an essential group of first-step producers in the terrestrial and marine biotopes. For example, TCS pollution has shown the highest sensitivity to algal species [21,22,23]. TCS has also been found to shift the composition of the biofilms forming in rivers, particularly the prokaryotic species, by killing a more significant proportion of the bacterial species compared to fungal and other species there [24,25]. With respect to more significant aquatic life, TCS has been found to impair the lipid metabolism of zebrafish embryos [26].

TCS’s mode of action as an antibacterial agent involves diffusion through the cell wall of the bacterial target and disruption of the cytoplasmic membrane, RNA, and lipid and protein synthesis of the bacteria [2,26]. TCS impairs the bacterial lipid synthesis through inhibition of the enoyl acyl carrier reductase of sensitive bacteria [7,11]. In studies involving rats, many adverse developmental effects have also been associated with TCS, including inhibition of circulating steroid hormone, disruption of fetal development [9], effects on thyroid hormone concentrations in male juvenile rats [27], damaging effects on hippocampal neuronal function, and impaired spatial memory in adult male rats [28]. While extensive research on the effect of TCS on humans has not yet been completed, relatively high-level concentrations of TCS have been found in urine, blood, and even breast milk [21]. Furthermore, studies on human cell cultures have indicated that TCS poses a risk of cell membrane damage and loss of mitochondrial transmembrane potential [29].

As mentioned earlier, several other phenolic compounds are ubiquitous in many industrial effluent streams since many of them are used as precursors for the manufacturing of various products ranging from biomaterials to petrochemicals [30]. Not surprisingly, as these phenolic compounds have similar functional groups as TCS, they exhibit similar types of negative effects in biological systems to those exhibited by TCS. As a result, they have raised a similar level of concern, warranting investigations of methods to remove them from aquatic systems as well.

Current techniques used for the removal of these compounds from water include electrochemical oxidation [31,32,33,34], adsorption by materials such as activated carbons [35,36], solvent extraction [37], photocatalytic degradation [38,39,40], advanced oxidations processes (AOPs) [33,41,42,43], and bioremediation using bacterial species and agricultural waste [42,43,44,45]. Among these methods, adsorption is particularly advantageous due to its ease of operation, both in batch and continuous processes, as well as the regeneration and reusability of the adsorbent materials. Photocatalytic degradation is interesting as well, especially since it allows for the decontamination of the chemical pollutants over a photocatalyst and since the photocatalyst can do so in many turn-over cycles before it loses its catalytic activity.

This article reviews some of the research progress made on the applications of mesoporous inorganic materials for the scavenging and photodegradation of TCS and related phenolic compounds such as phenol and phenol intermediates, which are detrimental to human health and the environment. The article focuses mainly on how adsorption and photocatalysis, as well as their combination, can be applied using mesoporous materials as platforms, with an emphasis on nanostructured silicas and hybrid materials composed of silica and titania, for the removal of these emerging pollutants from the aquatic systems. The unique properties, structures, and compositions of mesoporous nanomaterials suitable for the removal and decontamination of TCS and other phenolic compounds mentioned earlier are highlighted, along with some examples and the authors’ remarks. The examples in the paper are by no means comprhensive but rather illustrative of the points being highlighted. 

## 2. Nanomaterials for Adsorption of TCS and Phenolic Derivativess

Adsorption is widely used to salvage contaminants including TCS from water systems because adsorption processes are generally simple to design and apply and do not form harmful byproducts [46]. Adsorption is a surface phenomenon in which an adsorbate (a species in the gaseous or liquid phase) binds to the surfaces of a solid material called an adsorbent [47]. In practice, adsorption is performed using an adsorbent usually in powder form, either in a batch process or in continuous mode within columns. Under such circumstances, mass transfer effects are inevitable. The complete course of adsorption includes mass transfer and diffusion and comprises three main processes. The first two, which can be the limiting steps, involve film diffusion and mass transfer (on the external surfaces) or pore diffusion (on the internal surfaces of the system), and the last step includes surface adsorption via physical forces or electrostatic interactions (on the surfaces of the materials).

The literature is abundant with reports of various (nano)porous materials for adsorbing metal ions, dyes, and pollutants for environmental remediation applications [48]. The most widely explored such materials include metal oxides, such as MgO, SiO_2_, TiO_2_, and ZnO, and carbon-based materials. Most recently, other materials such as layered double hydroxides [43] and graphene oxides (GO) [34,49,50] have also been applied.

An array of synthetic methods is used to synthesize mesoporous nanomaterials such as SiO_2_, TiO_2_, and carbon-based materials. The creation of mesopores (or nanopores) inside these materials is a necessary step for creating effective adsorbents. These methods can be categorized as hard-templating, soft-templating, and template-free procedures.

The hard-templating method, also known as nano-casting, uses a solid template material to create the mesoporous structure. The template is then removed to leave behind mesopores inside the material. The templates include preformed nanoparticles, carbon, or mesoporous silica. One of the advantages of this technique is that there is less of a need to control the hydrolysis and condensation to help them favorably interact with the templates, which need to be considered when carrying out soft template-assisted co-condensation or self-assembly methods [51]. One of the drawbacks of this technique, especially compared with soft-templating method, is the non-availability of versatile hard templates with controllable structures and sizes, making the synthetic process time-consuming and less tailorable.

The soft-templating method uses soft matter or self-assembling substances that can serve as templates to produce the mesoporous structures in the materials. This method, for example, involves co-assembling surfactant molecules, such as Pluronic-123 and silicate guest species, to form an ordered mesoporous silica structure. This process can be performed in aqueous and nonaqueous solutions (i.e., hydrothermal and solvothermal or evaporation-induced self-assembly (EISA) processes, respectively) [52]. The template is then removed by either calcining the materials (typically above 350 °C to remove any organic components for at least 3 h) or solvent extraction (using an acidic solution or organic solvents). Calcination is one of the most common methods to remove soft templates due to the ease of operation and complete removal of the organic surfactant. However, its drawbacks include the complete loss of surfactants and the reduction in the density of surface hydroxyl groups on the material. The surface hydroxyl groups are vital for anchoring other functional groups on the surfaces of the material for many applications, including environmental remediation. The performances of mesoporous nanomaterial platforms for the removal of pollutants are also influenced by factors such as surface area, pore size, pore volume, and crystallinity. This, in turn, depends on the properties of the nanostructured platforms. To properly create and use nanomaterials for such applications, it is crucial to have a thorough grasp of their properties, which are radically different from those of their bulk counterparts.

While titania and carbon materials have been studied for the removal of emergent pollutants, including drugs with a rising prevalence in water systems [17], the number of reports on nanoporous and nanostructured adsorbents for TCS and phenolic compounds, and derivatives is still relatively less. On the other hand, these substances are increasingly becoming ubiquitous and posing significant environmental concerns, thus needing greater attention.

Generally, two approaches have been considered to remove these phenolic compounds or their derivatives from water by using mesoporous nanomaterials: adsorption and photocatalysis. In terms of adsorption-based extraction of TCS and other phenolic compounds from solutions, carbon materials have been widely studied. They were also among the first materials to be explored for this purpose. Carbon materials possessing good adsorption capacity for TCS can be made by designing their structures well enough to be able to adsorb this compound efffectively. For example, Yokoyama et al. investigated the ability of CO_2_-activated porous carbon that was derived from stevia residue for the adsorption of TCS [53]. Their tests showed that the material had a high surface area of 874 m^2^ g^−1^ and an adsorption capacity of 117 mg g^−1^ for TCS. Furthermore, their work indicated that the ability of the material to adsorb TCS varied with the pH of the solution as well as the surface area of the material. Because of its high adsorption capacity and the inexpensiveness of its precursor, CO_2_-activated porous carbon is a promising alternative material for the removal of TCS from solutions.

An excellent overview of the details of adsorption kinetics for contaminants in aquatic systems, along with the rate-limiting variables associated with the processes, is reported by Tan et al. [47]. Readers with interest in detailed information on this topic are referred to that article. Generally, two types of interactions are possible between an adsorbent and an adsorbate (TCS, in this case): physisorption (the attachment of adsorbate on adsorbent through weak attractive forces such as van der Walls interactions) and chemisorption (the attachment of adsorbate on adsorbent through the transfer or sharing of electrons between the two). These two processes can entail different adsorption kinetics depending on the adsorbent and adsorbate. Nevertheless, chemisorption is undesirable when the adsorbent needs to be reused in many cycles, which is the case in many adsorption processes for the extraction of contaminants such as TCS and phenolic compounds from the environment.

There have been differing views about the order of kinetic models involved in the adsorption of compounds on various adsorbents though. While numerous environmental studies have adopted pseudo-second-order models for adsorption processes involving pollutants such as TCS [47,54,55], other studies indicated that the adsorption and degradation of TCS with materials such as TiO_2_ nanoparticles could be described with a first-order kinetic model [56]. Nevertheless, the rate constants obtained by various models should be taken cautiously since lumped rate constants have no clear kinetic significance due to their unclear kinetic regimes [47]. Unfortunately, though, there have still been relatively few studies that describe the kinetics between adsorbates, such as TCS, and adsorbents since most of them have focused on materials synthesis and adsorption capacities rather than kinetics [57,58,59]. Additionally, kinetic studies to describe the adsorption of TCS and other phenolic compounds in the presence of other common species in solution [60], a situation close to a real waste stream system, are lacking.

## 3. Degradation of TCS and Related Phenolic Compounds

### 3.1. Formation of Carcinogenic Intermediates

One of the main challenges with the photodegradation of TCS is the formation of intermediates that can be more harmful in aquatic systems than TCS itself. For example, during photolysis, TCS can become 2,8-dichloro-dibenzo-p-dioxin, a carcinogenic substance [40,61]. In the environment, TCS can also undergo biological methylation and form methylated TCS, which is a more lipophilic and bioaccumulative compound than TCS [7]. Methylated TCS can thus affect living organisms more than TCS itself. Hence, photocatalytic systems that can fully degrade TCS to less harmful carboxylic acid products are desirable to prevent the transformation of TCS into more toxic compounds. Furthermore, carboxylic acid compounds are useful products that can be used for various purposes.

### 3.2. Effect of Solution pH

TCS is an ionizable compound, with pKa = 7.8, that can exist in two different forms in solutions depending on the pH of the solution. The neutral form (which is often referred to as HTric) exists mainly at pH < 7.8, while the anionic form (which is often referred to as Tric^−^) is present at pH > 7.8. The effects of pH on TCS’s ionization as well as degradation pathways in solutions under different conditions have been studied by various groups. For example, Solá-Gutiérrez et al. reported that the anionic form of TCS (pH > pKa: 7.9–8.1) would absorb light more readily than its neutral counterpart [40]. Lowering the pH below around 6.0 can thus reduce or not affect the rate of photolytic or photocatalytic degradation of TCS under such conditions. Note that the photolytic and photocatalytic degradation of TCS in solutions with different pHs could unfortunately also lead to harmful intermediates or products [24,62,63].

**Figure 3:**
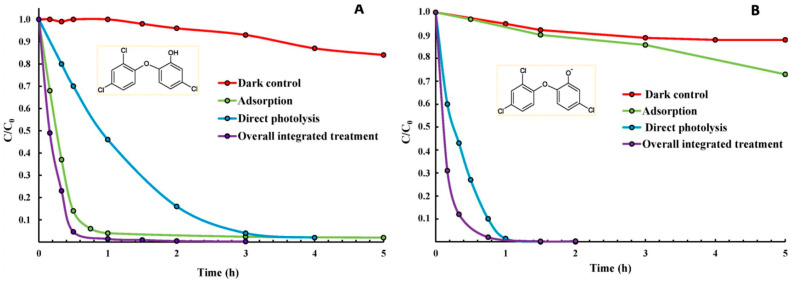
Concentration versus time curves showing the removal of TCS (C_0_ = 350 μg L^−1^) under direct photolysis or in the presence of the integrated photosensitizing adsorbent material in: (**A**) ultra-pure water (pH = 5.6) and (**B**) alkaline conditions (pH = 10.9). Control experiments that are performed under dark and control adsorption results are also included. Reproduced with permission from ref. [69].

Given that the pH in waste streams can vary, it is therefore also essential to design alternative mitigation methods that enable TCS to undergo the right course of photodegradation processes in solutions with different pH values. One such method involves the incorporation of photosensitizers that can help the photocatalytic conversion of TCS into harmless intermediates or products, as demonstrated by Marazuela et al. [64]. The authors specifically used Ru-based photosensitizers supported on polydimethylsiloxane (PDMS) membranes to photodegrade TCS (Figure 3) [64]. The system was found to oxidize and convert TCS by producing singlet oxygen (^1^O_2_) as reactive oxygen species (ROS), rather than the •OH species that had been proposed in multiple photodegradation pathways in many other systems [5,65]. It is worth adding that ROS-based oxidation can operate across a broader range of pH, unlike the •OH species-driven processes that can perform poorly in neutral and acidic conditions [62]. Thus, such ROS-based photocatalytic systems have potential applications for the treatment of pollutants such as TCS in a more comprehensive pH range in wastewater streams. The team also detected no harmful intermediates arising from the degradation of TCS including dioxins and chlorophenols. However, it should be noted that photosensitizers such as Ru-based dyes can be costly and susceptible to photobleaching [66]; thus, they are not viable for practical applications on a large scale. This begs for the development of other cost-effective and sustainable photosensitizers that can produce ROS-based species for the degradation of TCS and related compounds without producing toxic byproducts in the widest possible pH range. As an example, reduced TiO_2−x_ nanomaterials that can serve as highly effective and stable photocatalysts and that can operate both under UV and visible light have recently been developed by our group and others [67,68]. Such stable photocatalytic nanomaterials with long-lived charge carriers may have the potential to degrade organic compounds such as TCS. However, more research in this area, especially toward understanding their photophysical structure–photocatalytic activity relationships, as well as toward increasing their surface-active sites for degradation of pollutants, is still needed.

### 3.3. Effects of Competing Species on the Degradation of TCS and Related Compounds

The photodegradation of TCS can be hindered by various competing species in solution. For example, if present in solution, 2-propanol inhibits the photodegradation of TCS [69,70]. This is largely due to the competition between TCS and 2-propanol for the hydroxyl radicals (•OH) formed in the solutions under light. In addition, other species that can be present in the solution can compete with TCS for the photons in the light. Such substances, which are called photo-scavengers, include methanol, beta-carotene, and furfuryl alcohol. Due to their competition for the photons, these substances can inhibit the photodegradation of TCS, by up to 70% [40]. Thus, materials that are capable of efficiently degrading TCS even in the presence of competing substances for oxidative species or light (photo scavengers, hole scavengers, etc.), which are likely to be present in wastewaters containing TCS, should be developed.

## 4. Photocatalysis

### 4.1. Mesoporous Hybrid Materials

Hybrid nanomaterials, which are comprised of two or more components, have been reported to have numerous applications, including in photocatalysis and environmental remediation/protection [71,72,73,74,75]. Since such materials can be designed to possess the best properties of two or more materials, or because their combination can create synergistic effects, their overall properties could be better than those of the constituents. This includes their activities in photocatalysis as well as their ability to adsorb pollutants.

Hybrid nanomaterials can be created by various synthetic techniques using different materials as platforms or by systematically mixing different components. Owing to their low cost and environmental friendliness, titania-based materials such as commercially available P25 TiO_2_ are suitable platforms to deposit other species on and thereby create such hybrid materials for photocatalysis applications [76]. However, the photocatalytic efficiency of these materials is generally low because of their poor ability to absorb light and produce charge carriers that are required for the redox reactions involved in manyphotodegradation processes. In other words, the photoactivity of TiO_2_ is limited to the ultraviolet region of light (λ ≤ 390 nm) because it cannot absorb the visible and infrared regions of sunlight—the most readily available sources of energy from the Sun—to photocatalyze reactions. Furthermore, its synthesis is complicated by the fact that the rapid hydrolysis and condensation of its precursors often result in non-porous materials with poorly defined structures, low surface areas, and low density of catalytically active sites. Therefore, finding alternative synthetic methods [77] or alternative materials [78,79,80] that enable the synthesis of hybrid materials with good structures and photocatalytic properties to decontaminate TCS and other phenolic compounds is also of growing interest.

To this end, Brigante et al., for example, showed that hybrid mesoporous materials composed of TiO_2_–SiO_2_ can remove, via both adsorption and photocatalysis, the antibiotic tetracycline (a phenolic compound, which has become an emerging phenolic pollutant) more effectively than can pure TiO_2_ [81]. Furthermore, they showed that the adsorption capacity of the hybrid materials (e.g., 28 wt.% TiO_2_–SiO_2_) for this phenolic compound at a given pH was higher than that of TiO_2_. This is mainly because the hybrid materials have higher surface areas as well as more homogeneously dispersed TiO_2_ nanocrystallites than does pure TiO_2_. For instance, the hybrid material composed of 28 wt.% TiO_2_–SiO_2_ could photodegrade 60 µM of tetracycline in aqueous solution with 60% higher performance than the same amount of bare TiO_2_ could do. Thuis, such hybrid materials can serve as excellent platforms for both adsorption and photocatalytic degradation of tetracycline.

The incorporation of silica, especially in nanostructured and nanoporous form, into photocatalytic active materials is also proven to improve their applications in photocatalysis rendering the hybrid materials effective photocatalyts for the remediation of environmental pollutants. The nanostructured/nanoporous silica makes this possible by helping create large surface areas and controlled/tunable nanopore sizes in the hybrid materials [82]. For example, Wang et al. synthesized solid and hollow dendritic mesoporous silica-titania hybrid materials that showed better photocatalytic activities than the constituents [83]. The incorporation of silica into the titania nanomaterials allowed the titania nanoparticles to firmly anchor onto the surfaces of silica through strong Si-O-Ti cross-linked structures, reducing the titania nanoparticles’ tendency to aggregate. As a result, the hybrid mesoporous nanoparticles exhibited greater stability, due also to their increased wall thickness [84]. Furthermore, the larger pores make the adsorption of bigger molecules into the hybrid nanoparticles feasible [85] while dramatically increasing the mass transfer kinetics within their pores [46]. As a result, the photocatalytic decomposition processes of the molecules inside these nanoparticles take place better.

The structures of the materials can also affect how selective as well as how effective the photocatalytic properties of the hybrid materials will be towards specific pollutants. For example, hollow TiO_2_ nanomaterials was found to photodegrade organic dyes more selectively than commercial P25 TiO_2_ (Degussa) does, even if the former exhibited less adsorption capacity than their solid counterparts [83]. In another report, Gholizadeh et al. showed that SBA-16 type mesoporous silica would enable supported TiO_2_ (TiO_2_/SBA-16) to exhibit photocatalytic activity for degradation of phenol than pure TiO_2_ [58]. While a commercially available P25 TiO_2_ (Degussa) photodegraded only 27% of phenol in solution in 5 h, 10% TiO_2_/SBA-16 enabled the removal of 62% phenol in solution in the same period under the same experimental conditions. While in this work, phenol was studied as a model pollutant, it could serve as a framework for exploring the potential photocatalytic application of such materials for the degradation of TCS and other common phenolic emerging pollutants in the same way [58]. More importantly, these examples highlight the design of photocatalytic materials with the right structures and compositions to improve their photocatalytic activity and selectivity toward the degradation of specific organic pollutants from phenolic contaminants from water systems.

### 4.2. Functionalized Nanoporous Hybrid Materials

Functionalizing the surfaces of nanoporous materials with different moieties can improve the ability of the materials to adsorb and remove pollutants such as TCS from aqueous solutions. For example, Toufaily et al. synthesized organic-functionalized SBA-15 mesoporous silica using organosilanes that contain thiol and amine groups to improve the material’s selectivity to adsorb and extract phenolic compounds from aqueous systems [86]. The adsorption capacity of the materials was found to be linearly related to the number of amine groups on their surfaces. Additionally, their surface amine groups, which are hydrophilic, allowed them to disperse well in aqueous solutions and to serve as adsorbents better. Meanwhile, the thiol groups on the surfaces of the particles helped them exhibit selective adsorption properties towards the semi-hydrophobic TCS. Overall, the chemical modification of nanoporous adsorbents with different functional groups is a viable synthetic strategy to produce nanoporous materials with enhanced adsorption capacity as well as selectivity for various phenolic pollutants, including TCS [87,88,89].

### 4.3. Mesoporous Metals and Metal Oxide-Supported Metals for Photodegradation of TCS and Other Phenolic Compounds and Their Derivatives

Incorporating monometallic nanoparticles and bimetallic nanoalloys into nanoporous support materials, e.g., semiconducting metal oxides, is another promising strategy for producing nanostructured photocatalysts for emerging pollutants. The nanoparticles can constitute favorable electronic and structural properties and synergistic effects that enable effective photocatalysis. Noble metal nanoparticles can do so, for example, by absorbing light via surface plasmonic resonance (SPR) or by stabilizing the charge carriers (electrons and holes) forming on the semiconducting metal oxides upon light illumination. Additionally, metal nanoparticles can serve as electron traps, thereby reducing the recombination of the charge carriers on semiconducting metal oxides and improving their chances to transfer to adsorbates to degrade them. Furthermore, the immobilization of metals on the surfaces of other materials can create photocatalytic groups that enable the decomposition of adsorbates/pollutants [90]. The immobilization metallic nanoparticles into other materials can also prevent/minimize their leaching into groundwater systems, which is an important parameter for public safety and health when such materials are considered for practical applications.

To this end, various metallic nanomaterials have been integrated with photocatalytically active systems to improve the performances of the photocatalysts. For instance, Belekbir et al. incorporated/doped different amounts of different metals (namely, Cu, Cr, and V) into nanosized TiO_2_ and then investigated the effects of the metals on the materials’ photocatalytic performances under UV and near UV-Vis light irradiation using phenol as a model pollutant [70]. In the case of Cu-doped TiO_2_, 0.5% Cu-doped TiO_2_ was found to degrade phenol faster than commercial P25 TiO_2_ catalyst in the first 30 min. However, the metal-doped TiO_2_ could not fully degrade phenol in the UV-Vis region. Interestingly though, all synthesized metal-impregnated photocatalysts showed more efficient and faster kinetics in removing phenol in the near UV-visible region compared to P25-TiO_2_. Their photocatalytic activities were different from each other though. The sizes of the crystallites and the surface area of the materials were ruled out from being the main factors in the differences in the activity of these photocatalytic materials since the sizes of the particles in the three catalysts were comparable. A relevant factor was proposed to be the fact that metal dopants act as hole (h^+^)- and/or electron (e^−^)-trapping sites, which, in turn, impede the rate of electron/hole pair recombination, thereby increasing the ability of the charge carriers to participate in the redox processes during photocatalysis.

**Figure 4:**
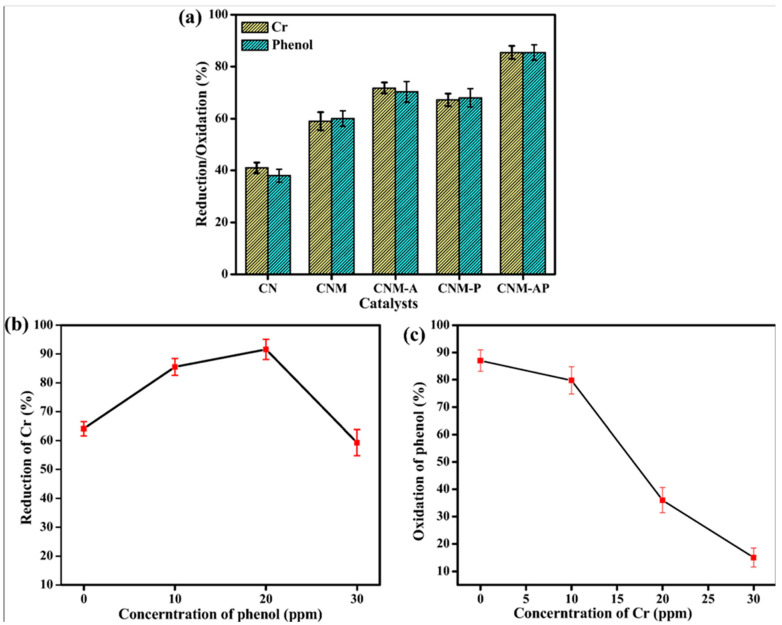
(**a**) Percent reduction of Cr(VI) ions and oxidation of phenol over different catalysts. (**b**) Reduction of Cr(VI) ions in the presence of varying concentrations of phenol. (**c**) Oxidation of phenol in the presence of varying concentrations of Cr(VI) ions. The experiments are performed in triplicates. The sample names are: CN = carbon nitride; CNM = carbon nitride/MCM-41 mesoporous silica; CNM-A = Au modified CNM; CNM-P = Pd-modified CNM; and CNM-AP = Au-Pd-modified CNM. Reproduced with permission from ref. [91].

Similar mechanisms entailing the roles of metal dopants in photocatalytic systems were reported by Patnaik et al. [91]. In their work, the authors demonstrated the favorable effects that alloyed metal dopants (Au-Pd) provide to mesoporous silica (MCM-41)-modified g-C_3_N_4_, rendering the hybrid material photocatalytic active toward environmentally relevant reactions. Of note, they showed that the material enables photocatalytic oxidation of phenol and hexavalent chromium (Cr(VI)), another ubiquitous pollutant, individually as well in tandem (Figure 4). The removal of Cr(VI) and phenol in tandem was noticeably faster when catalyzed by Au-Pd-modified MCM-41/g-C_3_N_4_ (denoted CNM-AP) in comparison to the one catalyzedt by Au-modified or Pd-modified MCM-41/g-C_3_N_4_ (denoted CNM-A and CNM-P, respectively). However, the rates of removal of phenol via oxidation over the materials decreases as the concentration of Cr(VI) increases (Figure 4b,c). Furthermore, the authors showed that under acidic conditions (e.g., pH~5.1), the presence of 20 ppm phenol sped up the rate of the reaction reducing Cr(VI) to about 91.6% from an initial concentration of 20 ppm. However, a lower concentration (10 ppm) of phenol caused a reduction of Cr(VI) by 85.1% from a solution with the same initial concentration. The group suggested that organic compounds (phenol, in this case) would assist the photocatalytic degradation of the metallic pollutants (Cr(VI) species) by serving as electron donors. This means that organic compounds (or pollutants) can enhance the rate of degradation of other pollutants by improving charge separation processes while they are undergoing photodegradation themselves. Furthermore, the work demonstrated that the removal of two pollutants from contaminated water efficiently and simultaneously without using any additional electron/hole scavengers would be possible.

As mentioned previously, the presence of phenolic intermediates must be accounted for when designing photocatalytic systems since the intermediates themselves can also exhibit toxicity. Thus, photocatalytic systems that drive the total oxidation of the organic pollutants are more desirable. For the system discussed above, for example, the intermediate aromatic compounds were oxidized further via a ring-opening reaction into CO_2_ and H_2_O while assisting the removal of Cr(VI) species [90]. Another interesting finding by the group was that, in the presence of light, phenol scavenges the photogenerated holes faster than the Cr(VI) species do. This allows the phenol to restrict the recombination of the electrons with holes on the surfaces of the catalyst, accelerating the reduction of Cr(VI) by the photogenerated electrons. Therefore, a sort of one-two punch is achieved where both pollutants undergo their respective degradation. A drawback to this system is the high cost of the metal dopants such as Au and Pd used to functionalize the mesoporous systems. Therefore, many other groups have been exploring inexpensive and techno-economically viable alternatives that are scalable for photocatalytic degradation of emerging pollutants [46,92,93].

**Figure 5:**
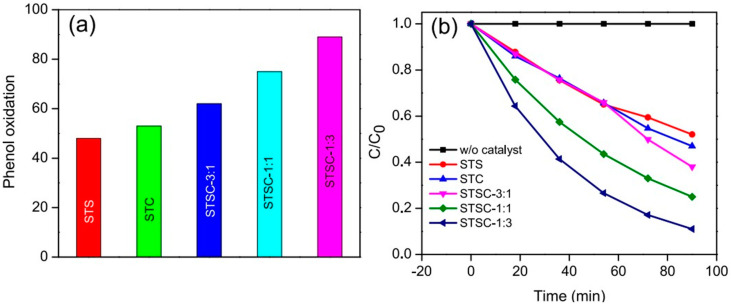
(**a**) Catalytic performances of different core-shell SiO_2_@TiO_2_ photocatalysts functionalized with Ag, Cu, or both for phenol oxidation. (**b**) The relative concentration of phenol in solution is degraded by different core-shell SiO_2_@TiO_2_ photocatalysts functionalized with Ag, Cu, or both. The sample names are: STS = silica-titania (SiO_2_@TiO_2_) core-shell microspheres that are modified with silver; STC = silica-titania core-shell microparticles that are modified with copper; STS = SiO_2_@TiO_2_ nanoparticles that are modified with copper; STSC = silica-titania core-shell microparticles that are modified with silver and copper in different ratios; e.g., STSC-3:1, represents 3:1 mol ratio of Ag-to-Cu). Reproduced with permission from ref. [73].

In another interesting recent work, Babu and Naik synthesized Cu and Ag bimetallic nanoalloys-decorated SiO_2_@TiO_2_ microspheres and then demonstrated their photocatalytic properties under visible light for the degradation of phenol [73]. They also showed that the materials would photocatalyze the water reduction reaction to produce hydrogen (H_2_) under the same conditions. The nanomaterials were synthesized by anchoring Ag and Cu alloy nanoparticles onto SiO_2_@TiO_2_ core-shell microspheres through multistep synthetic processes involving sol-gel and chemical reduction methods. The authors also tested the photocatalytic properties of the materials synthesized with different ratios of Cu/Ag (Figure 5). Their results indicated that the incorporation of Cu and Ag improved the materials’ ability to absorb visible light. Notably also, the core-shell SiO_2_@TiO_2_ photocatalyst containing Ag-to-Cu in 1:3 mol ratio (or STSC-1:3, where the numbers in the sample’s notation represent the ratio of Ag-to-Cu) resulted in a higher reaction rate for the degradation of phenol than those containing other ratios of Ag-to-Cu. The degradation of phenol via oxidation on these materials is attributed to the participation of hot holes generated by the bimetallic nanoalloys and the holes generated on TiO_2_. These holes participate in the reaction better and enhance the material’s photocatalytic activity because the energy barrier formed at the metal alloys/TiO_2_ interface suppresses the electron-hole recombination, leaving most of the holes available for the degradation reaction.

**Figure 6:**
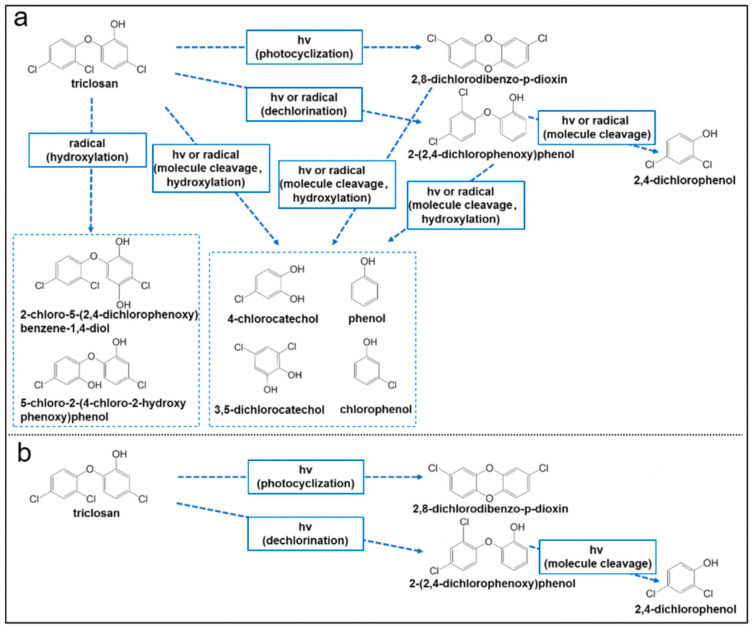
Various possible photodegradation pathways for TCS over metal oxide (silica) nanoparticles in (**a**) suspensions and (**b**) in the form of particles. Reproduced with permission from ref. [94].

It is worth adding here that the photocatalytic processes involving TCS and other related phenolic compounds over various metal oxides could also be inhibited by various unwanted processes. Although the metal oxide particles help produce ROS that promote the photodegradation of TCS in suspensions, they could prevent light from reaching all the reactive sites on the materials. This could, in turn, reduce the photodegradation of TCS and other phenolic compounds over the materials. Furthermore, the photodegradation of TCS and the other phenolic compounds by metal oxides could produce secondary pollutants in the environment by forming other toxic intermediates or byproducts, as illustrated in Figure 6 below [94].

### 4.4. Multifunctional Photocatalysts

**Figure 7:**
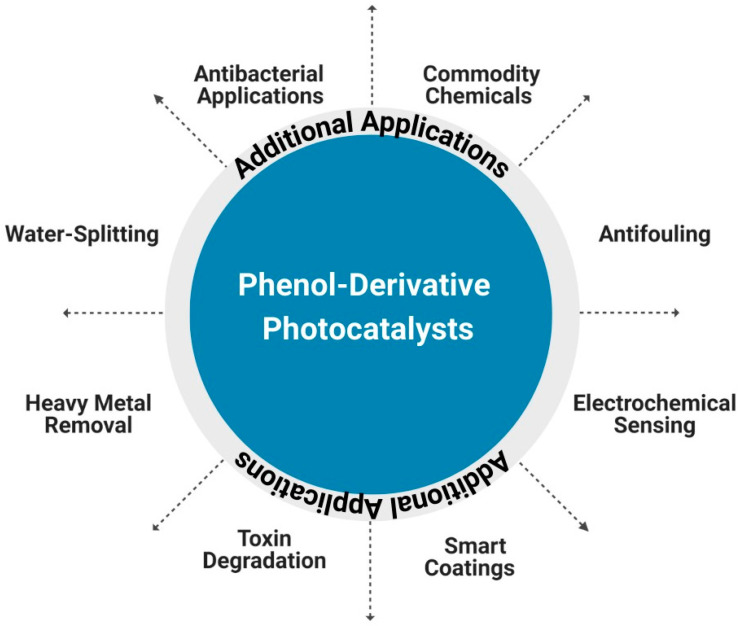
Phenol-degrading photocatalysts can perform additional functions based on various reports found in the literature.

Another aspect to consider when designing photocatalysts for the degradation of emerging phenolic compounds is to have them perform additional functions. This means a photocatalyst used for the degradation of phenol and related species can be designed to have also antibacterial, antifouling, or anticorrosive properties for environmental protection. Several research groups have recently ventured into this area and obtained promising results. Figure 7 shows a snapshot with examples of such multifunctional materials, which can degrade phenolic compounds and their derivatives while also performing secondary functions, for example, in aquatic systems.

For example, Ferreira et al. synthesized a visible-light-driven TiO_2_ photocatalyst co-doped with cobalt and nitrogen for the degradation of TCS [95]. An interesting additional function of the material is its ability to serve as an antimicrobial agent against both gram-positive and gram-negative bacteria [95]. Notably, the photocatalyst had an increased effect against *Legionella pneumophila*, a common pathogen found in aquatic environments. These two properties that this material exhibits can make this material suitable for water treatment where both TCS or other phenolic species and pathogens are present.

In a complementary line of work, Coelho et al. developed a photocatalytically active zirconia-titania ultrafiltration membrane that can photodegrade phenol and humic acid under simulated sunlight irradiation [96]. As a secondary function, the membrane is shown to have self-cleaning or antifouling properties. These multi-functional properties can be useful in industrial settings by reducing (or perhaps eliminating) the need for post-treatment of effluents, reducing operational costs, and minimizing the water-purification process footprint.

**Figure 8:**
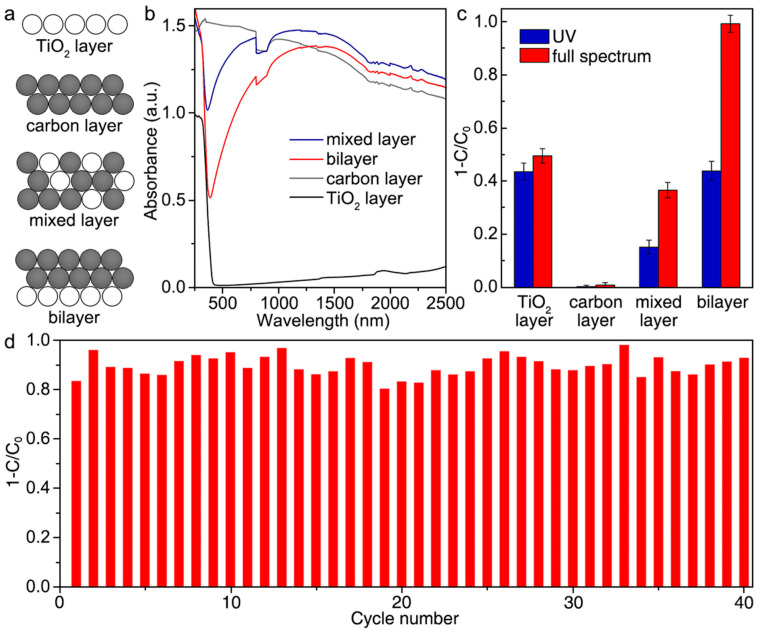
(**a**) Diagrammatic representation of TiO_2_ layer, carbon (C) layer, TiO_2_/C mixed layer, and TiO_2_/C bilayer that are tested for photocatalysis. (**b**) Diffuse reflectance spectra of the materials. (**c**) Photocatalytic properties of the materials for the degradation of phenol under irradiation with UV and full spectrum of light. (**d**) Recyclability test results for TiO_2_/C bilayer paper under several photocatalytic cycles indicating its stability. Reproduced with permission from ref. [97].

**Figure 9:**
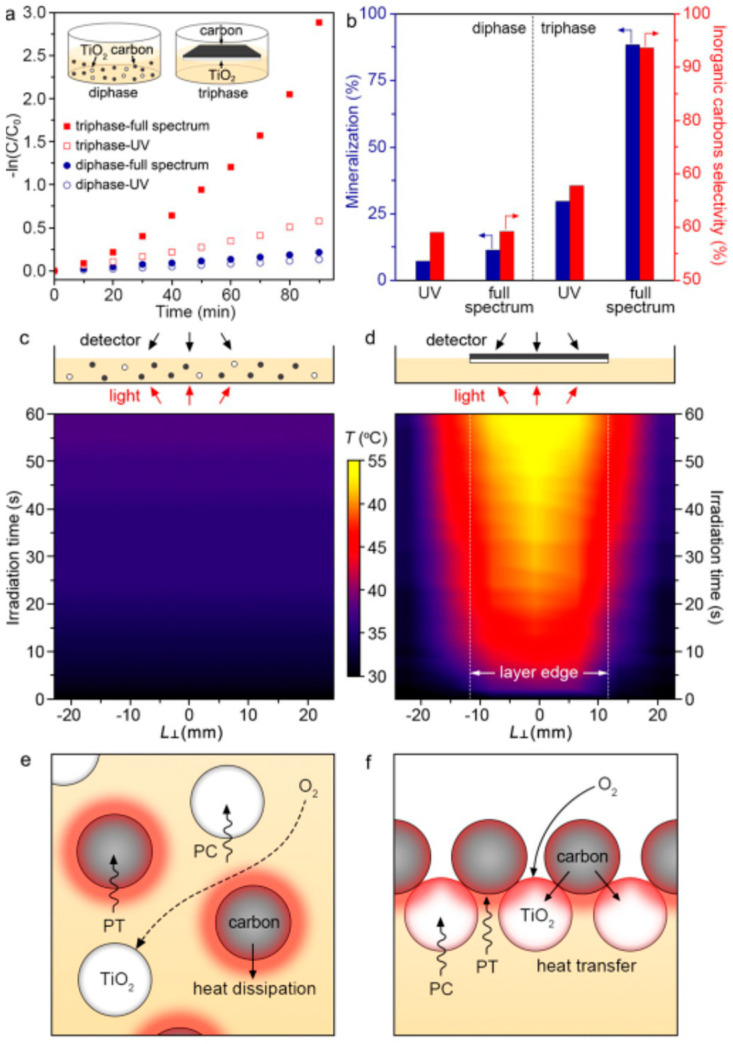
Photocatalytic activity evaluation of diphase and triphase systems. (**a**) Pseudo-first-order kinetic curves of the photocatalytic decomposition reaction involving phenol over diphase and triphase systems. Inset in (**a**) schematically shows the setup of the two photocatalytic systems. (**b**) Percentages of phenol mineralization and selectivity to inorganic carbons during photocatalytic decomposition of phenol under different light sources. Time-dependent, temperature distribution diagram of (**c**) diphase system and (**d**) triphase system. Schematic illustration of oxygen diffusion and photothermal effect in (**e**) diphase system and (**f**) triphase system. In the last two figures, PC represents photocatalysis and PT represents photothermal effect. Reproduced with permission from ref. [97].

Huang et al. developed a bilayer catalytically active paper composed of TiO_2_ and carbon (C) materials to degrade phenol via photothermal-assisted triphase photocatalysis [97]. Their work also demonstrated the distinct advantages such a multifunctional catalyst in the form of “paper” had over dispersions of powder and photoelectrode systems for photodegradation of phenolic and related compounds. Notably, the catalyst is selective towards phenol over hydroquinone and p-benzoquinone during photocatalysis while maintaining its activity in over 40 cycles. These qualities, coupled with its scalable synthesis, make this multifunctional catalyst a promising candidate for the selective degradation of pollutants within a flow reactor. Figure 8 and Figure 9 show the material’s photocatalytic performances under UV and full spectrum of light as well as its recyclable use as a catalyst and some of its characterization results.

Table 2 shows additional examples of multifunctional photocatalytic materials reported in the literature both for the removal of TCS/phenolic compounds and for other (secondary) applications. It should be noted that for the scope of this review, only platform materials consisting of SiO_2_, TiO_2_, or both are considered and included in the table. Note that these materials are doped with other elements to bring about (good) photocatalytic activities or other desirable secondary functionality. These materials are chosen among many others for illustrative purposes.

## 5. Conclusions and Future Outlooks

TCS and other phenolic compounds and their derivatives are becoming ubiquitous in the environment, especially in aquatic systems, and are causing a significant impact there due to their wide applications in various consumer products. While there have recently been increased reports in the literature on their potential negative side effects, more research is necessary to find ways to remove these emerging pollutants from the environment. It is also important to understand the adsorption kinetics and mechanisms involving such emerging pollutants in various systems in order to design and fabricate materials that can helo with the removal of these compounds from the environment. Equally important, new materials to effectively capture remove, and decontaminate these pollutants, even before finding their way into aquatic systems and water systems, must be developed. In this review, the unique properties, structures, and compositions of silica- and titania-based nanomaterials being developed for the removal and photocatalytic decontamination of TCS and other phenolic compounds from the environment are discussed. While several materials have been considered and explored for these purposes, mesoporous silica and titania are among the most notable ones. These two materials are viable platforms for the adsorption of these compounds from the environment because of their robust structures, recyclability, and potential for anchoring other functional groups to increase their adsorption affinity for these compounds. In addition, their pore sizes and structures can be tuned to tailor their adsorption capacity for these pollutants as well as to study the adsorption kinetics between the pollutants and the materials. Some of them can also photocatalyze the decontamination of TCS and various other phenolic compounds and enable the studies of the photocatalytic processes and mechanisms associated with these emerging environmental pollutants. Some of the works performed in this area include the development of various photocatalytic materials for the degradation of these compounds. One commonly applied process involves the conversion of TCS and phenolic compounds to harmless byproducts using a catalytic or photocatalytic component incorporated within the pores of the mesoporous materials. However, further research into the development of highly active, selective, and recyclable nanostructured photocatalysts able to perform such conversions is still currently needed.

Most recently, there has also been a push to develop multifunctional materials that can degrade TCS and other phenolic pollutants in the environment via photocatalysis as well as that can address other problems typically associated with water remediation operations such as fouling, bacterial contamination, or persistent organic pollutants. Since such materials can potentially perform multiple functions at the same time, they are of greater interest for applications in the decontamination of water systems.

There have been advances in the stability and recyclability of these materials, but additional research is needed to scale up and deploy them in existing operations. Other aspects to consider are the logistics and potential for recovering and reusing these materials once the adsorption and photocatalytic processes are carried out. These issues should be addressed before any industrial-scale implementation of these various nanomaterials is developed for decontamination of the environment from TCS and other ubiquitous phenolic compounds.

## Figures and Tables

**Table 1:** Selected regulatory agencies around different geographical regions of the world that make decisions on the use of triclosan (TCS) in consumer products and their recent decisions regarding TCS, including completely banning its use in various products.

Geographic Region ^a^	Regulatory Agency	Use and Limitations of TCS	Ref.
European Union	Scientific Committee on Consumer Safety (SCCS)	Banned from use as a biocidal product Restricted use as a preservative in consumer products: Toothpaste; hand soaps; body soaps/shower gels; deodorants (non-spray); face powders and blemish concealers; nail products for cleaning fingernails and toenails before the application of artificial nail systems—with a maximum concentration of 0.3%. Mouthwashes—with a maximum concentration of 0.2%.	[12]
USA	Food and Drug Administration (FDA)	Companies must undergo a premarket review to be able to use TCS in over-the-counter (OTC) consumer antiseptic products. Unregulated use in hand sanitizers, wipes, and other cleaning products.	[6,13,14]
Latin America	Mexican Secretariat of Health	Set at 0.5% in all products.	[15]
Japan	Pharmaceuticals and Medical Devices Agency (PMDA)	Set at ≤0.1% in all products.	[13]
South Africa	South African Health Products Regulatory Authority (SAHPRA)	The tolerable concentration of products containing TCS is set at approximately between 0.2 and 0.3%.	[16]
South Korea	Korean Food and Drug Administration (KFDA)	Rinse-off: body cleansing products, deodorant (excluding spray products). Used locally: foundation to hide skin defects (e.g., blemish concealers) at ≤0.3%. TCS is prohibited from use in toothpaste or mouthwash.	[13]

^a^ These countries are selected mainly because data on TCS from there are available in the literature as well as to balance the geographical representation of different parts of the world while conveying the information.

**Table 2:** Summary of versatile applications of phenol derivative-degrading photocatalytic materials.

Platform (and Nanomaterial)	Target Pollutant	Additional Functionality	Highlights	Ref.
TiO_2_ (Cobalt and nitrogen co-doped TiO_2_ anatase nanoparticles)	TCS	Antibacterial against *Legionella pneumophila*	The material degrades > 99% TCS in 20 min from 10 ppm solution under UV and light emitting diode (LED) light. It can also serve as an antibacterial agent against *Legionella pneumophila*, *Staphylococcus* aureus (https://www.sciencedirect.com/topics/medicine-and-dentistry/staphylococcus accessed on 4 September 2023), and *Escherichia* coli (https://www.sciencedirect.com/topics/medicine-and-dentistry/escherichia accessed on 4 September 2023)	[95]
SiO_2_@TiO_2_ (SiO_2_@TiO_2_ core-shell nanomaterials)	Phenol	Water-splitting	AgCu (in 1:3 mol ratio) deposited on core-shell SiO_2_@TiO_2_ hybrid nanomaterials for phenol oxidation and photocatalytic hydrogen generation under visible light. Compared with the monometallic materials, the hybrid material shows two-times stronger catalytic activity toward phenol oxidation and three-times higher photocatalytic activity toward hydrogen generation while producing eight-times greater photocurrent.	[73]
SiO_2_ (Core-Shell SiO_2_@Ag NCs@Ag_3_PO_4_)	Phenol	Water-splitting and phenol oxidation	The material catalyzes water splitting and phenol oxidation up to 91% in 120 min. During water splitting, it catalyzes the hydrogen evolution reaction and the oxygen evolution reaction with rates of 2460 mol h^−1^ g^−1^ of H_2_ and 1236 mol h^−1^ g^−1^ of O_2_, respectively.	[98]
TiO_2_ (TiO_2_/g-C_3_N_4_ as photocatalyst)	Phenol	Photocatalyst for degradation of dyes, phenol, and caffeine.	Under UV light irradiation, the material effectively photocatalyzes the degradation of methylene blue and caffeine, with methylene blue degradation reaching nearly 100% after 240 min and phenol degradation reaching 75% after 300 min.	[99]
SiO_2_ (Bismuth oxychloride/mesoporous silica)	Phenol	Antibacterial, heavy metal stripping analysis.	The composite material shows photocatalytic activity toward the degradation of rhodamine B as well as strong antibacterial activity against *Staphylococcus aureus* and *Enterococcus faecalis*. However, the material’s activity for phenol degradation is not as effective as other materials reported in the literature.	[100]
TiO_2_ (Ce-Y-ZrO_2_/TiO_2_ on ZrO_2_/SiC support material fabricated as a membrane)	Phenol, humic acid	Antifouling or self-cleaning	The membrane is effective for photodegrading phenol and humic acid under simulated sunlight irradiation. The membrane also exhibits better anti-fouling (smaller flux decline) and higher permeation flux properties under irradiation compared to filtration in the dark. Moreover, the membrane shows self-cleaning properties upon irradiation, which enables recovery of up to 97% of the original flux. The experiment using commercial TiO_2_ (P25) results in 100% phenol degradation in 150 min. Although the unsupported membrane (Ce-Y-ZrO_2_/TiO_2_) shows a lower activity, resulting in 70% degradation, it is easier to recover and reuse. The fact that the membrane can easily be separated from water systems after being used makes it also more advantageous.	[96]

## Data Availability

No new data were created or analyzed in this study. Data sharing is not applicable to this article.
